# Effects of background color and restoration depth on color adjustment potential of a new single-shade resin composite versus multi-shade resin composites

**DOI:** 10.3389/fbioe.2023.1328673

**Published:** 2023-12-07

**Authors:** Jiakang Zhu, Siyang Chen, Annikaer Anniwaer, Yue Xu, Cui Huang

**Affiliations:** State Key Laboratory of Oral and Maxillofacial Reconstruction and Regeneration, Key Laboratory of Oral Biomedicine Ministry of Education, Hubei Key Laboratory of Stomatology, School and Hospital of Stomatology, Wuhan University, Wuhan, China

**Keywords:** color adjustment potential, resin composites, color shifting, single shade, CIEDE2000

## Abstract

The objectives of this study was to evaluate the effects of background color and restoration depth on color adjustment potential of a new single-shade resin composite versus multi-shade resin composites. Two multi-shade resin composites (Spectrum TPH3 and Clearfil AP-X) marked A2 shade and a new single-shade resin composite (Charisma Diamond One) were tested. Four base shades (A1, A2, A3, and A3.5) of the same resin composite (Filtek Z250) were selected as different background colors. Dual specimens with 1-, 2-, and 3-mm restoration depth and single specimens of all materials were fabricated. CIE color coordinates were measured using a spectrophotometer, then color differences (∆E_00_) and translucency parameter (TP_00_) were calculated using the CIEDE2000 formula. Independent observers performed visual scoring. CAP-I and CAP-V values were calculated according to ΔE_00_ and visual scoring. The results revealed that CAP-I and CAP-V were significantly affected by resin composite type, background color, and restoration depth. CAP-I and CAP-V decreased as restoration depth increased at the same background color for all materials. Charisma Diamond One had the highest CAP-I and CAP-V values at all background colors and restoration depths, with the highest TP_00_ value. These findings demonstrated that color adjustment potential was dependent on resin composite type, background color, and restoration depth, so shade selection is indispensable for multi-shade resin composites. Charisma Diamond One exhibited the highest color adjustment potential and the most pronounced color shifting, contributing to simplifying the process of shade selection and improving the efficiency of clinical work.

## 1 Introduction

Resin composites are now widely used as esthetic restorative materials in dentistry, which can perfectly achieve the restoration of tooth defect and the modification of tooth color and shape in a conservative and inexpensive way (Korkut and Türkmen, 2021; Da Rosa Rodolpho et al., 2022). Unlike indirect restorations made of ceramic materials whose color can be achieved by external staining at the later stage of treatment, the accurate shade selection is necessary for resin composite restorations before treatment, so as to select the materials closest to the color of the surrounding teeth for good esthetic effects (Ismail et al., 2020; Diamantopoulou et al., 2021; Vargas et al., 2023). The shade of resin composite selected based on a light-cured resin ball refers to placing a resin ball on the surface of tooth, comparing the color difference between the tooth and the resin ball after light-curing, and then removing it until a resin composite with the appropriate shade is determined by clinicians. Obviously, the shade selection of resin composite is complex and time-consuming (Browning et al., 2009; Tabatabaian et al., 2021).

In clinic, we can feel that the color differences between resin composites and surrounding tooth tissues are less perceived when viewed as a whole after finishing the restorations than when viewed separately before filling treatment (Tsubone et al., 2012; de Abreu et al., 2021). This phenomenon is called “chameleon effect” in dental parlance interpreted as color shifting which includes two major aspects: the blending effect (not measurable by any instrument, an optical illusion) and the effect of physical translucency (Paravina et al., 2008; Ismail and Paravina, 2022). More recently, color adjustment potential (CAP) is a term that describes and quantifies the interaction between the physical and perceptual components of color shifting, which can be assessed both instrumentally (CAP-I) and visually (CAP-V) (Trifkovic et al., 2018; Pereira Sanchez et al., 2019). Previous studies have shown that the CAP of resin composite is affected by many factors such as the type and shade of resin composite, background color, restoration depth, etc. (Tanaka et al., 2015; Akgül et al., 2022; El-Rashidy et al., 2022; Yamashita et al., 2023).

In order to minimize the shade selection, simplify the process of resin composite restoration, and reduce the chair-side time, single-shade resin composites were created and introduced, which have only a narrow range of colors but can match the colors of different teeth (de Abreu et al., 2021; Lucena et al., 2021; Altınışık and Özyurt, 2023). In other words, single-shade resin composites have enhanced CAP.

Recently, a newly developed single-shade resin composite (Charisma Diamond One, Kulzer, Hanau, Germany) has been introduced, which can supposedly match all Vita Classical shades according to the manufacturer’s information (Altınışık and Özyurt, 2023). However, no published third-party study has evaluated the CAP of the new single-shade resin composite compared with multi-shade resin composites to truly prove its superiority in color matching over conventional multi-shade resin composites.

Therefore, this study aimed to evaluate and compare the instrumental and visual color adjustment potential (CAP-I and CAP-V) of the newly developed single-shade resin composite compared with two clinically available multi-shade resin composites in relation to background color and restoration depth. The null hypotheses tested were: (1) there are no significant differences in color adjustment potential (CAP-I and CAP-V) among the resin composites evaluated, (2) background color or restoration depth would have no effect on the color adjustment potential (CAP-I and CAP-V).

## 2 Materials and methods

### 2.1 Specimen preparation

Two multi-shade resin composites marked A2 shade and a new single-shade resin composite were evaluated in this study. Four base shades (A1, A2, A3, and A3.5) of the same resin composite were selected to simulate different background colors. Details of these materials were listed in Table 1.

**TABLE 1:** Materials used in this study.

Groups		Materials	Shade	Manufacturer	Composition	Lot number
Test	T1	Clearfil AP-X	A2	Kuraray Noritake, Okayama, Japan	Base resin: Bis-GMA, TEGDMA	5C0133
					Filler: silanated barium glass filler, silanated silica filler, silanated colloidal silica	
	T2	Spectrum TPH3	A2	Dentsply, Konstanz, Germany	Base resin: Bis-GMA, Bis-EMA, TEGDMA	2111000345
					Filler: barium aluminio borosilicate, barium fluoro aluminio borosilicate, highly dispersed silicon dioxide	
	T3	Charisma Diamond One	-	Kulzer, Hanau, Germany	Base resin: TCD-Urethaneacrylate, UDMA, TEGDMA Filler: Barium Aluminium Boro Fluor Silicate Glass	K010026
Base	B1	Filtek Z250	A1	3M ESPE, St. Paul, MN	Base resin: Bis-GMA, Bis-EMA, UDMA Filler: zircon/silica	NE03476
	B2		A2			NE62695
	B3		A3			NE03675
	B4		A3.5			NA26526

Bis-EMA, ethoxylated bisphenol A glycol dimethacrylate; Bis-GMA, Bisphenol A diglycidylmethacrylate; TCD-Urethaneacrylate, Tricy clodecane-Urethaneacrylate; TEGDMA, triethylene glycol dimethacrylate; UDMA, urethane dimethacrylate.

**FIGURE 1:**
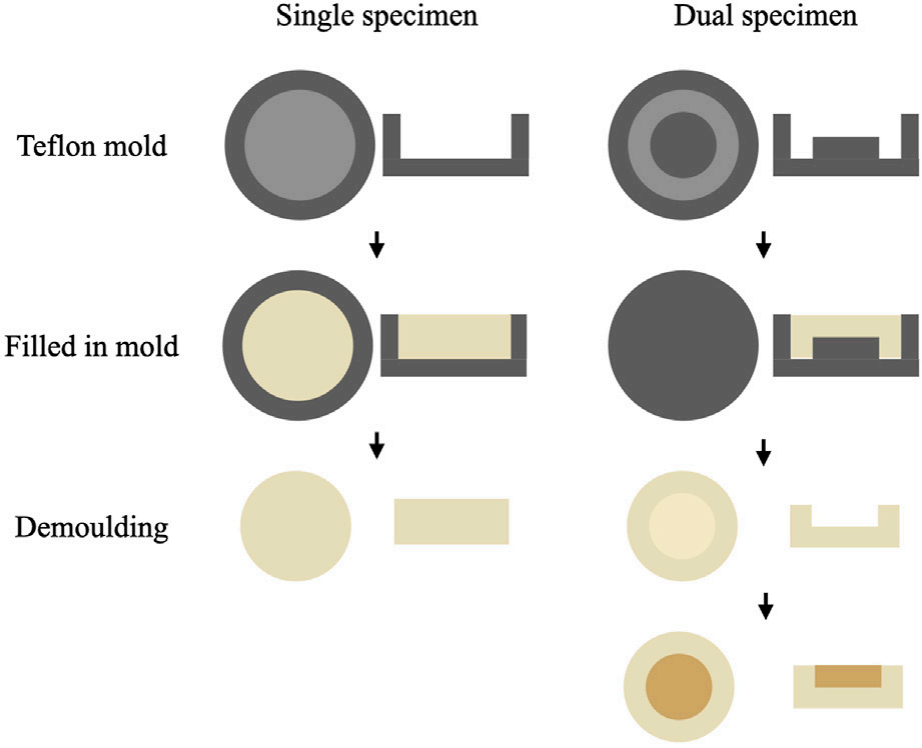
Diagram for the fabrication of single and dual specimens. The heights of raised part in the hole of Teflon molds for dual specimens were 1.0 mm, 2.0 mm, and 3.0 mm respectively in different groups.

**FIGURE 2:**
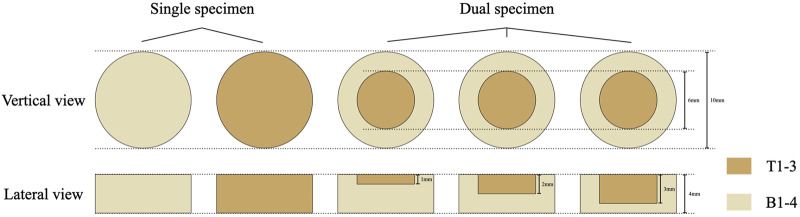
Schematic representation of single and dual specimens.

All specimens were made in custom-designed, Teflon molds (Figure 1). All specimens were filled in twice and each time filled the materials with a thickness of approximately 2 mm and then light-cured for 20 s using an LED light curing unit (Bluephase II; Ivoclar Vivadent, Schaan, Liechtenstein). The resin composite specimens were divided into four groups: three groups of dual specimens and one group of single specimens (Figure 2). The single specimens were disk-shaped (diameter = 10 mm, thickness = 4 mm) and fabricated for all materials included in this study (*n* = 9). The dual specimens consisted of an outer ring (diameter = 10 mm, thickness = 4 mm) made of base shades and an inner hole in the center (diameter = 6 mm) filled with each of the 3 tested materials after the cavities were treated with a transparent universal bonding agent (3 M-ESPE, St Paul, MN, United States) (n = 9). to According to the depth of the inner hole (corresponds to the thickness of the tested shade), dual specimens were divided into 3 groups including 1.0 mm-group, 2.0 mm-group, and 3.0 mm-group for each test material (Akgül et al., 2022; Yamashita et al., 2023).

The same operator progressively polished all specimens on both sides using wet silicon carbide paper of increasing grit number (P600-, P800-, and P1200-grit) for 10 s each, at a speed of 150 rpm, with mild hand pressure in a table-top grinder-polisher (EcoMet 250; Buehler, Lake Bluff, IL, United States). All specimens were incubated at 37°C for 24 h to ensure complete polymerization.

### 2.2 Instrumental evaluation

A portable clinical spectroradiometer (Vita Easyshade V; VITA Zahnfabrik, Bad Sackingen, Germany) was used for the color measurements for all specimens against both black and white backgrounds. The probe (diameter = 5 mm) of spectroradiometer faced the center of the specimen during measurement. The spectrophotometer was calibrated in compliance with the manufacturer’s instructions before each measurement. L^*^, a^*^, and b^*^ color coordinates based on the CIELAB system were recorded, where L^*^ represents the lightness on a scale of 0 (black) to 100 (white), a^*^ represents the hue and chroma on the red-green axis, and b^*^ represents the hue and chroma on the yellow-blue axis.

Color coordinates measured were used for computation of color differences. The color difference (ΔE) was calculated using the CIEDE2000 color difference formula as follows (Perez Mdel et al., 2011):

${\Delta E}_{00}=\sqrt{{\left(\frac{{\Delta L}^{\prime }}{K_{L}S_{L}}\right)}^{2}+{\left(\frac{{\Delta C}^{\prime }}{K_{C}S_{C}}\right)}^{2}+{\left(\frac{{\Delta H}^{\prime }}{K_{H}S_{H}}\right)}^{2}+R_{T}\left(\frac{{\Delta C}^{\prime }}{K_{C}S_{C}}\right)\left(\frac{{\Delta H}^{\prime }}{K_{H}S_{H}}\right)}$ , where ΔL′, ΔC′, and ΔH' were the differences in lightness, chroma, and hue for a pair of points. S_L_, S_C_, and S_H_ were the weighing functions for the lightness, chroma, and hue components, respectively. The parametric factors (K_L_, K_C_, and K_H_) were the expressions for experimental conditions. All parametric factors of the CIEDE2000 color difference formula were set to 1. R_T_ was the rotation factor that considers the interactions between hue and chroma differences in the blue area.

In addition, translucency parameter (TP_00_) values were determined by calculating the color coordinates values between the readings over the black and white backgrounds according to the following CIEDE2000 color difference formula (Lucena et al., 2021):

${\text{TP}}_{00}=\sqrt{{\left(\frac{L_{B}^{\prime }-L_{W}^{\prime }}{K_{L}S_{L}}\right)}^{2}+{\left(\frac{C_{B}^{\prime }-C_{W}^{\prime }}{K_{C}S_{C}}\right)}^{2}+{\left(\frac{H_{B}^{\prime }-H_{W}^{\prime }}{K_{H}S_{H}}\right)}^{2}+R_{T}\left(\frac{C_{B}^{\prime }-C_{W}^{\prime }}{K_{C}S_{C}}\right)\left(\frac{H_{B}^{\prime }-H_{W}^{\prime }}{K_{H}S_{H}}\right)}$ , where the subscripts “B” and “W” referred to the lightness (L′), chroma (C′) and hue (H′) of the specimens over the black and white backgrounds, respectively.

### 2.3 Visual evaluation

Visual color evaluations were performed by three dentistry specialists with demonstrated superior color discrimination competency according to ISO/TR 28642:2016. Under D65 illumination and using a 0°/45° viewing geometry, the observers performed blind visual evaluations of all specimens placed on a neutral gray paper in a random order. Color differences were graded from 0 to 4 as follows: 0 = excellent match, 1 = very good match, 2 = not so good match (border zone mismatch), 3 = obvious mismatch, and 4 = huge (pronounced) mismatch.

### 2.4 Color adjustment potential (CAP) indices

**FIGURE 3:**
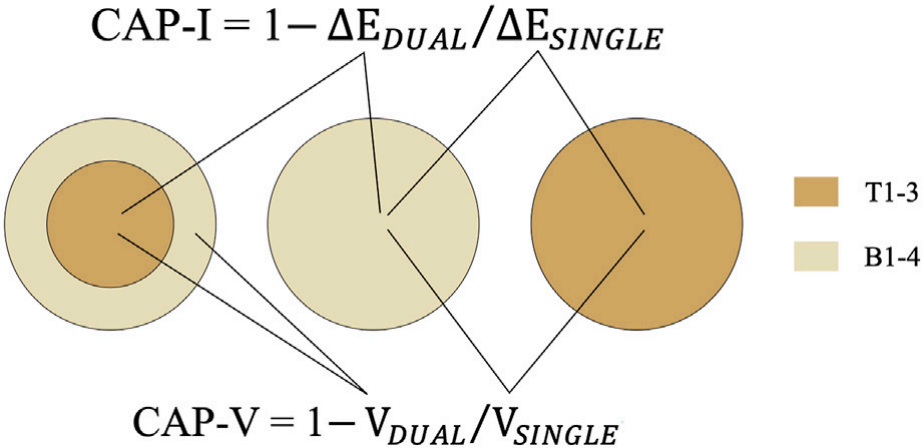
The measurement areas of instrumental and visual evaluation; the calculation method of CAP-I and CAP-V.

The CAP indices included instrumental CAP (CAP-I) index and visual CAP (CAP-V) index (Figure 3).

CAP-I was calculated as follows: CAP-I = 1 $-{\Delta E}_{DUAL}/{\Delta E}_{SINGLE}$ , where ΔE_DUAL_ was the CIEDE2000 color difference between the test shade in dual specimen and the base shade both in single specimen, and ΔE_SINGLE_ was the CIEDE2000 color difference between the test shade and base shade in separate single specimen.

CAP-V was calculated as follows: CAP-V = 1 $-V_{DUAL}/V_{SINGLE}$ , where V_DUAL_ was the visual scoring between the test shade and base shade both in the same dual specimen, and V_SINGLE_ is the visual scoring between the test shade and base shade in separate single specimen.

### 2.5 Statistical analysis

Statistical analysis was performed with IBM SPSS Statistics for Mac (version 26; IBM Corp., Armonk, NY, United States). The data were presented as mean and standard deviation values. The result of the Shapiro–Wilk test showed that the data presented normal distribution (*p* > 0.05). A three-way analysis of variance (ANOVA) was used to analyze the effects of material type, background color, and restoration depth on ∆E_00_, CAP-I, and CAP-V values. The least significant difference (LSD) test and Dunnett’s T3 test were applied for multiple comparisons when significant variation was detected. The test standard was bilateral (α = 0.05 for all tests).

## 3 Results

**FIGURE 4:**
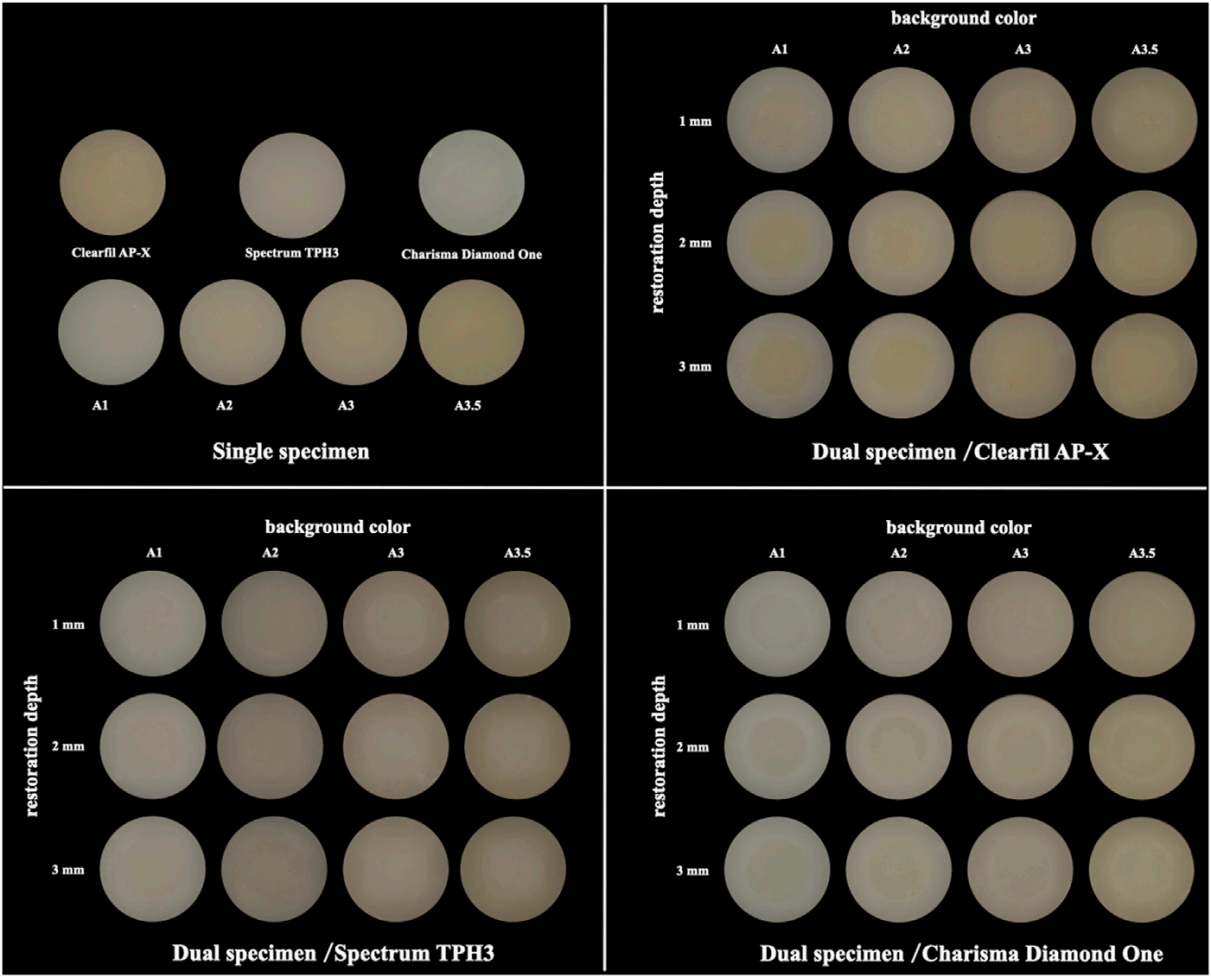
The representative single and dual specimens of all test materials and base materials.

Figure 4 showed representative single and dual specimens. In order to eliminate unwanted specular reflections on the surfaces of specimens caused by the flash, the photos were taken with a cross-polarization filter, which contributed to improving color selection and communication (Villavicencio-Espinoza et al., 2018; Sampaio et al., 2019; Jorquera et al., 2022).

The mean and standard deviation values of ∆E_00_ and visual scoring for the single specimens and the dual specimens with three restoration depths were respectively shown in Tables 2, 3. According to the results of multiple comparisons, ∆E_00_ values and visual scoring were both significantly affected by material type, background color, restoration depth, and specimen type (*p* < 0.001).

**TABLE 2:** Descriptive statistics and multiple comparisons of ∆E_00_ values.

Material	Background color	Single specimen	Dual specimen (Restoration depth)		
			1 mm	2 mm	3 mm
Clearfil AP-X	A1	7.80 ± 0.74^A,a,*^	5.19 ± 0.67^A,b^	6.92 ± 0.59^A,a^	7.38 ± 0.55^A,a^
	A2	5.53 ± 0.42^B,a^	4.09 ± 0.35^B,b^	4.83 ± 0.43^B,c^	5.57 ± 0.63^B,a^
	A3	3.08 ± 0.37^C,a^	1.97 ± 0.23^C,b^	2.91 ± 0.39^C,a,*^	4.85 ± 0.52^C,c,*^
	A3.5	2.65 ± 0.12^D,a^	2.02 ± 0.15^C,b^	2.52 ± 0.21^C,a^	3.85 ± 0.55^D,c^
Spectrum TPH3	A1	4.42 ± 0.75^A,a^	2.63 ± 0.27^A,b^	3.72 ± 0.58^A,a^	4.18 ± 0.57^A,a^
	A2	2.43 ± 0.26^B,a^	1.70 ± 0.16^B,b^	1.87 ± 0.17^B,b^	2.61 ± 0.23^B,a^
	A3	2.24 ± 0.50^B,a^	2.34 ± 0.36^C,a^	2.81 ± 0.38^C,b,*^	3.32 ± 0.57^C,c^
	A3.5	4.97 ± 0.57^A,a^	4.15 ± 0.28^D,b^	5.32 ± 0.36^D,a,c^	5.60 ± 0.26^D,c^
Charisma Diamond One	A1	7.89 ± 0.65^A,a,*^	1.65 ± 0.29^A,b^	1.84 ± 0.22^A,b^	2.76 ± 0.45^A,c^
	A2	8.56 ± 0.51^A,a^	2.69 ± 0.19^B,b^	2.92 ± 0.19^B,b,c^	3.06 ± 0.13^A,c^
	A3	8.28 ± 0.31^A,a^	4.19 ± 0.44^C,b^	4.40 ± 0.27^C,b^	5.31 ± 0.74^B,c,*^
	A3.5	9.88 ± 0.30^B,a^	4.76 ± 0.28^D,b^	6.05 ± 0.31^D,c^	7.22 ± 0.45^C,d^

The same superscript uppercase letter indicates no statistical difference among the background colors at the same material type and type/restoration depth of specimen (*p* > 0.05).

The same superscript lowercase letter indicates no statistical difference among the type/restoration depth of specimen at the same material type and background color (*p* > 0.05).

* indicates no statistical difference among the material types at the same background color and type/restoration depth of specimen (*p* > 0.05).

**TABLE 3:** Descriptive statistics and multiple comparisons of visual scoring.

Material	Background color	Single specimen	Dual specimen (Restoration depth)		
			1 mm	2 mm	3 mm
Clearfil AP-X	A1	3.82 ± 0.17^A,a,*^	1.93 ± 0.36^A,b^	2.18 ± 0.29^A,b^	3.04 ± 0.35^A,c^
	A2	2.48 ± 0.53^B,a^	1.48 ± 0.38^A,b,*^	1.56 ± 0.33^B,b,*^	2.18 ± 0.24^B,a^
	A3	1.74 ± 0.28^C,a,*^	0.89 ± 0.23^B,b,*^	1.26 ± 0.32^B,c,*^	1.78 ± 0.44^B,C,a,*^
	A3.5	1.52 ± 0.34^C,a^	0.93 ± 0.32^B,b,*^	1.37 ± 0.31^B,a^	1.55 ± 0.47^C,a^
Spectrum TPH3	A1	3.07 ± 0.36^A,a,#^	1.15 ± 0.17^A,B,b^	1.41 ± 0.33^A,b^	1.74 ± 0.22^A,B,c^
	A2	2.04 ± 0.35^B,a^	0.89 ± 0.33^A,b,#^	0.96 ± 0.39^B,b^	1.55 ± 0.29^A,c,*^
	A3	1.81 ± 0.34^B,a,*^	1.41 ± 0.33^B,b^	1.63 ± 0.26^A,a,b,c^	1.81 ± 0.38^A,B,a,c,*^
	A3.5	2.11 ± 0.41^B,a^	1.85 ± 0.34^D,a^	2.04 ± 0.26^C,a,*^	2.07 ± 0.36^B,a,*^
Charisma Diamond One	A1	3.41 ± 0.46^A,a,*,#^	0.81 ± 0.34^A,b^	1.07 ± 0.32^A,b,c^	1.37 ± 0.31^A,c^
	A2	3.82 ± 0.34^A,B,a^	1.19 ± 0.44^A,b,*,#^	1.48 ± 0.38^B,b,c,*^	1.70 ± 0.26^B,c,*^
	A3	3.96 ± 0.11^B,a^	1.04 ± 0.35^A,b,*^	1.22 ± 0.41^A,B,b,*^	1.74 ± 0.32^B,c,*^
	A3.5	4.00 ± 0.00^B,a^	1.15 ± 0.56^A,b,*^	2.00 ± 0.37^C,c,*^	2.15 ± 0.45^C,c,*^

The same superscript uppercase letter indicates no statistical difference among the background colors at the same material type and type/restoration depth of specimen (*p* > 0.05).

The same superscript lowercase letter indicates no statistical difference among the type/restoration depth of specimen at the same material type and background color (*p* > 0.05).

* or # indicates no statistical difference among the material types at the same background color and type/restoration depth of specimen (*p* > 0.05).

From the perspective of instrumental evaluation, for Charisma Diamond One, the ∆E_00_ values of all dual specimens were significantly lower than those of single specimens against the same background color. However, for Clearfil AP-X and Spectrum TPH3, the ∆E_00_ values of only a small portion of the dual specimens (mainly with 1-mm restoration depth) were significantly lower than those of the single specimens. But from the perspective of visual evaluation, the visual scorings of dual specimens were statistically significantly lower than or equal to those of single specimens at the same background color for all materials with different restoration depths. Nevertheless, for all materials, both ∆E_00_ values and visual scorings increased as restoration depth increased against the same background color.

In addition, at the same restoration depth, Clearfil AP-X showed the highest ∆E_00_ values and visual scorings against A1 base shade and the lowest mainly against A3/A3.5 base shade, Spectrum TPH3 showed the highest ∆E_00_ values and visual scorings against A3.5 base shade and the lowest mainly against A2 base shade, and Charisma Diamond One showed the highest ∆E_00_ values against A3.5 base shade and the lowest against A1 base shade.

**FIGURE 5:**
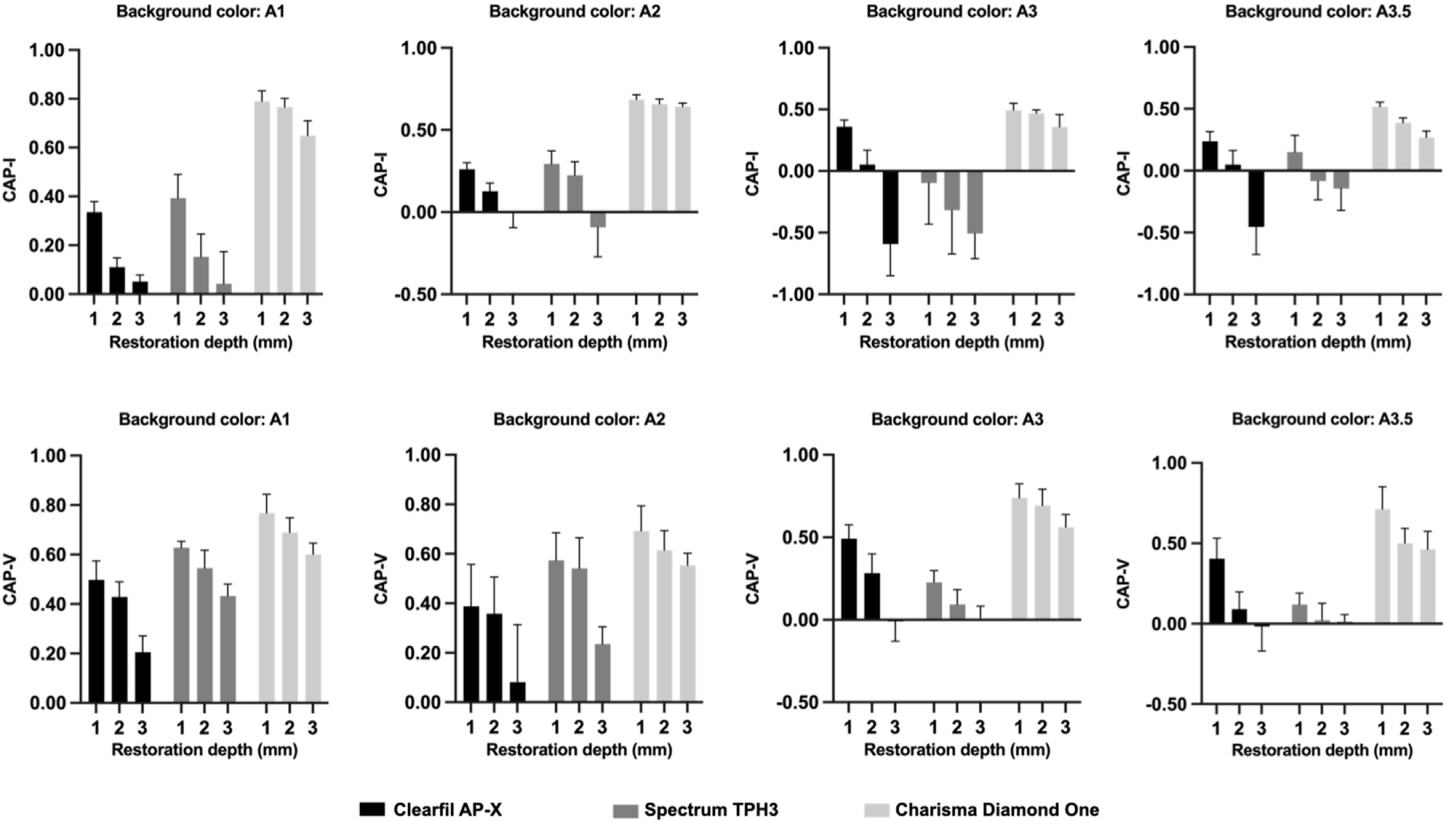
The CAP-I and CAP-V values of three test materials with different restoration depths under four background colors.

According to the three-way ANOVA results, CAP-I and CAP-V values were both significantly affected by material type, background color, restoration depth, and their interactions (*p* < 0.001) (Table 4). Figure 5 presented the CAP-I and CAP-V values of three test materials with three restoration depths under four background colors. For all materials, both CAP-I and CAP-V decreased as restoration depth increased against the same background color. CAP-I values ranged from −0.45 to 0.79 and CAP-V values ranged from −0.02 to 0.77, both with the highest values consistently found for Charisma Diamond One among three test materials at the same background color and restoration depth (*p* < 0. 001).

**TABLE 4:** Three-way ANOVA results of CAP-I and CAP-V.

Object	Source	Type III sum of squares	df	Mean square	F	*p*
CAP-I	Material type (A)	20.645	2	10.322	591.045	<0.001
	Background color(B)	6.459	3	2.153	123.286	<0.001
	Restoration depth (C)	6.675	2	3.337	191.093	<0.001
	A * B	1.094	6	0.182	10.439	<0.001
	A * C	1.549	4	0.387	22.172	<0.001
	B * C	0.750	6	0.125	7.159	<0.001
	A * B * C	1.320	12	0.110	6.299	<0.001
CAP-V	Material type (A)	9.147	2	4.573	437.432	<0.001
	Background color(B)	3.556	3	1.185	113.387	<0.001
	Restoration depth (C)	3.667	2	1.834	175.372	<0.001
	A * B	2.103	6	0.351	33.525	<0.001
	A * C	0.43	4	0.107	10.274	<0.001
	B * C	0.296	6	0.049	4.724	<0.001
	A * B * C	0.335	12	0.028	2.667	0.002

**FIGURE 6:**
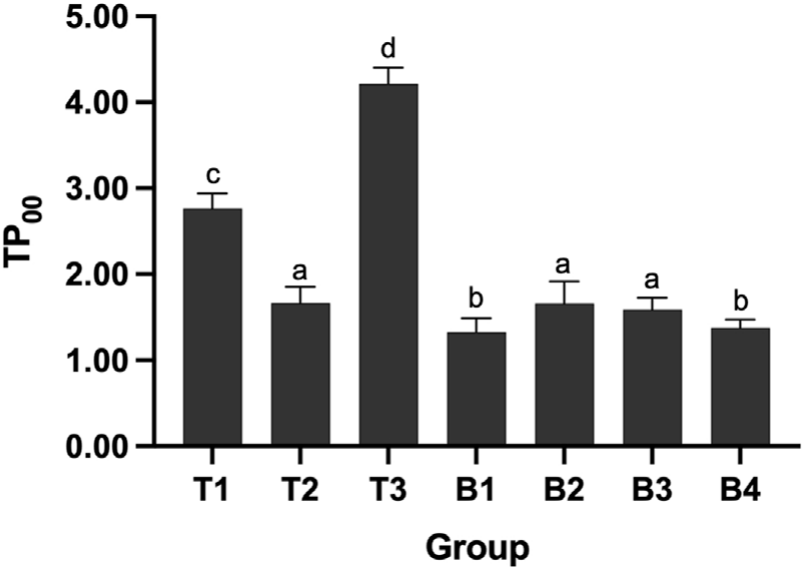
The TP_00_ values of all test materials and base materials for the single specimens. Different lowercase letters indicate the significant statistical difference (*p* < 0.05).

Figure 6 showed the TP_00_ values of all test materials and base materials for the single specimens. Charisma Diamond One and Filtek Z250 (A1 shade) showed, respectively, the greatest and the lowest translucency values, with significant differences (*p* < 0.001). There was no statistically significant difference among the TP_00_ value of Spectrum TPH3, Filtek Z250 (A2 shade), and Filtek Z250 (As shade) (*F* = 0.396, *p* = 0.678), as well as between the TP_00_ value of Filtek Z250 (A1 shade) and Filtek Z250 (A3.5 shade) (*F* = 0.597, *p* = 0.560).

## 4 Discussion

The results of present study showed significant differences in both instrumental and visual color adjustment potential among all test resin composites at different background colors and restoration depths. Therefore, both experimental hypotheses were rejected.

The color adjustment potential can be evaluated instrumentally and visually. Color measurement instruments can describe the results in terms of color coordinates. Spectrophotometers have been proven to be useful and accurate instruments for measuring color in dentistry, which can improve the sensitivity and accuracy of measurement compared with visual evaluation (Karamouzos et al., 2007; Johnston, 2009). However, visual evaluation remains an important indicator for evaluating the color difference and color adjustment potential of resin composites (Chu et al., 2010), as it is the most commonly used method primary basis for clinicians and patients to determine the esthetic effect of resin composite restoration in clinic (de Abreu et al., 2021; Ruiz-López et al., 2022). Therefore, both instrumental and visual evaluation were used in present study to comprehensively compare the color adjustment potential of different resin composites. The CIELAB formula is frequently-used in the evaluation of color difference in dentistry. However, in order to achieve a better correlation between visual perception and instrumental evaluation, the recent color difference formula CIEDE2000 was developed and increasingly popular (Pecho et al., 2016; Günal-Abduljalil and Ulusoy, 2022).

The result from the present study was in accordance with previous investigations that the color difference of resin composite restoration depended on the material type and background color (Pereira Sanchez et al., 2019; de Abreu et al., 2021; Saegusa et al., 2021). Because the types and contents of base resins and fillers, as well as the size and shape of fillers, all have an impact on the color shifting ability of resin composites. Previous studies founded a positive correlation between the amount of Bis-GMA in the resin composite and its translucency because Bis-GMA has a higher translucency compared to UDMA and TEGDMA (Azzopardi et al., 2009; Miletic et al., 2017). And some studies also showed the color shifting ability of resin composites would improve with the increase in the filler contents (Altınışık and Özyurt, 2023). In addition, the size and shape of resin composite fillers determine the final surface properties of restorations, such as the size and shape of defects on the surfaces after polishing, resulting in the different wavelengths reflected from the surface and affecting the human perception of color (Sang et al., 2021; da Costa et al., 2021). Although the shade of all test resin composites (except Charisma Diamond One) was selected as A2 shade in this study, not all materials showed the lowest color difference under background of A2 shade. In this study, Spectrum TPH3 marked A2 shade matches the Filtek Z250 marked A2 shade best, with the lowest ∆E_00_ values and visual scoring among four background colors. However, Clearfil AP-X marked A2 shade matches the Filtek Z250 marked A3 or A3.5 shade best. Therefore, shade selection is indispensable in clinic when using multi-shade resin composites, especially when it comes to highly esthetic restorations (Ismail et al., 2020). The shade selection does not just determine the shade of the tooth needed treatment. More importantly, the shade of the resin composite selected based on the light-cured resin balls is necessary, because there are color differences between different resin composites marked the same shade.

CAP is a term representing color shifting referring to the interaction of dental materials with surrounding tissues, which determine the esthetic effect of resin composite (Ismail and Paravina, 2022). Equally, CAP was also depended on the material type and background color. The resin composite with positive CAP value means that it has less color differences when viewed together with surrounding after restoration than when viewed separately (Durand et al., 2021). Currently, there has been no uniform standard for the CAP value as a threshold of effective color shifting yet. Pereira et al. and Altınışık et al. thought that a CAP value of 0.20 (corresponding to a 20% reduction in the value of color difference in dual specimen compared with single specimen) was the threshold (Pereira Sanchez et al., 2019; Altınışık and Özyurt, 2023), while Durand et al. thought a CAP value of 0.50 was the threshold (Durand et al., 2021). However, there is no doubt that among all the materials tested, Charisma Diamond One exhibited the best and effective color shifting ability at all different background colors and restoration depths, with CAP-I or CAP-V values always greater than 0.20 and the vast majority greater than 0.5.

As expected, the color difference and CAP were significantly affected by restoration depth. The color difference (both ∆E_00_ value and visual scoring) increased as restoration depth increased against the same background color for all test materials, while CAP-I and CAP-V values decreased as restoration depth increased. This result indicated that the esthetic effect of resin material was declined as restoration depth increased, which highlighted the importance of layering technique in esthetic restoration of resin composite. The color construction of natural tooth is more complex than the experimental sample, so layering technique is necessary to simulate the optical properties of a natural tooth and minimize the color difference when it comes to resin composite restoration, especially for the anterior teeth with high esthetic requirements (Romero, 2015; Miotti et al., 2017; Ricci and Fahl, 2023).

Previous studies have reported that the CAP of resin composite increased in accordance with an increase in translucency (Paravina et al., 2006). Translucency is one of the primary factors in controlling esthetics and it can be evaluated by the translucency parameter (TP_00_) (Lucena et al., 2021). The higher the TP_00_ value is, the more light can be reflected from the background color into the composite restoration (Wang et al., 2013). This may explain the higher CAP-I and CAP-V values of Charisma Diamond One were recorded compared to other resin composites, with the highest TP_00_ value among all materials.

According to the manufacturer and literature, Charisma Diamond One utilizes “adaptive light matching” which determines the shade of the restoration by absorbing light waves reflected from the surrounding tooth shade. It contains urethane methacrylates, whose refractive index decreases as the size of side alkyl chain increases. Therefore, the translucency of Charisma Diamond One could increase after curing (Altınışık and Özyurt, 2023). On the other hand, due to the effect of the dark background in the oral cavity, the level of translucency that is incompatible with the surrounding structures can result in grayish restorations for the anterior teeth (Lucena et al., 2021). This also explains the higher ∆E_00_ value and visual scoring were recorded for Charisma Diamond One compared to other resin composites. One possible solution to this problem may be the “blocker”, which is used for the restoration of anterior teeth, so as to better combine the resin composite with the adjacent tooth structure (de Abreu et al., 2021).

Apart from the CAP, color difference is also an important indicator for evaluating the color shifting ability of resin composite. Some previous studies have proposed to adjust the acceptable threshold of color difference to ΔE_00_ ≤ 1.8 units (Paravina et al., 2015). According to this standard, only Spectrum TPH3 with 1- and 2-mm restoration depths under A2 background color and Charisma Diamond One with 1- and 2-mm restoration depths under A1 base shade displayed the acceptable color difference. However, ΔE00 value is affected by measuring instrument, illuminant condition, environment brightness, background color, and so on (Lagouvardos et al., 2009; Lee et al., 2010; Tabatabaian et al., 2021). The result of visual evaluation showed that most of the tested groups of the dual specimens were superior or equal to “border zone mismatch” (visual scoring ≤ 2). Combined with the result of visual evaluation, the acceptable threshold value of ΔE00 in this study should be higher than 1.8.

Based on the comprehensive results of this study, Charisma Diamond One had the best color shifting ability, for its better CAP at different background colors and restoration depths. Charisma Diamond One is likely to match different teeth colors, which contributes to simplifying the process of shade selection and improving the efficiency of clinical work. However, it may only be suitable for restoration of the posterior teeth. For the restoration of the anterior teeth with high esthetic requirements, its application may be limited by the fact that the color difference evaluated instrumentally or visually was not minimal all the time for it and it may result in grayish restorations.

The current study has several limitations. Only four background colors were evaluated and the specimen cannot completely simulate the color construction of a natural tooth. And the experimental conditions for color evaluation are different from clinical treatment. In addition, the color stability of resin composites can be significantly affected by the aging process in the oral environment such as changes in temperature and the absorption of colorants contained in foods and beverages, which affects the esthetic result and clinical longevity of resin composite restorations. The evaluation of these variables needs to be further studied *in vivo* and *in vitro*, which will provide more detailed information about the color properties of resin composites.

## 5 Conclusion

The color adjustment potential was significantly affected by resin composite type, background color, and restoration depth, regardless of instrumental or visual evaluation. Charisma Diamond One exhibited the most pronounced color shifting ability with the highest CAP-I and CAP-V values at all background colors and restoration depths, compared to other multi-shade resin composites. These findings demonstrated that shade selection is indispensable for multi-shade resin composites and Charisma Diamond One contributes to simplifying the process of shade selection and improving the efficiency of clinical work.

## Data Availability

The original contributions presented in the study are included in the article/Supplementary Material, further inquiries can be directed to the corresponding author.
